# Significant glomerular IgM deposition predicts poorer kidney outcomes in lupus nephritis compared with other forms of immune complex deposits

**DOI:** 10.1136/lupus-2025-001708

**Published:** 2025-10-10

**Authors:** Wang Xiang, Yaoyao Tang, Xiuzhi Jia, Yuewen Lu, Xinxin Zhang, Xiaolei Shi, Jianwen Yu, Hongjian Ye, Zhong Zhong, Jiang Lanping, Xi Xia, Ruihan Tang, Wei Chen

**Affiliations:** 1 Department of Nephrology, The First Affiliated Hospital of Sun Yat-sen University, Guangzhou, Guangdong, China; 2 NHC Key Laboratory of Clinical Nephrology (Sun Yat-Sen University) and Guangdong Provincial Key Laboratory of Nephrology, Guangzhou, Guangdong, China

**Keywords:** Lupus Nephritis, Antibodies, Risk Factors

## Abstract

**Objective:**

Glomerular immune complex deposition plays a central role in lupus nephritis (LN), but the prognostic relevance of individual immunoglobulin components remains unclear. This study aimed to investigate the clinical impact of glomerular immunoglobulin M (IgM) deposition intensity on patient outcomes.

**Methods:**

This retrospective cohort study analysed 952 biopsy-proven LN patients (1996–2019) from the First Affiliated Hospital of Sun Yat-sen University. A semiquantitative scoring system stratified glomerular immunoglobulin G (IgG), immunoglobulin A (IgA), IgM, complement 3 (C3) and complement component 1q (C1q) deposition into low (−/+) and high (++ to ++++) groups. The primary outcome was a composite of doubling of serum creatinine from baseline or the development of end-stage renal disease (ESRD). The secondary outcome was all-cause mortality. A multivariable Cox regression model was used to adjust for baseline clinical and pathological factors.

**Results:**

Among the studied immune complexes, only high glomerular IgM deposition was significantly associated with adverse renal outcomes (p=0.025). These patients had higher baseline Systemic Lupus Erythematosus Disease Activity Index scores (SLEDAI) (16 (12–20) vs 15 (12–18), p<0.001), more severe histopathological features (including proliferative glomerulonephritis, endocapillary hypercellularity, leucocyte infiltration and microthrombi), and profound complement activation (lower median serum C3 and complement 4 (C4) levels, both p<0.05). High glomerular IgM deposition also correlated with high IgA (r=0.48) and C3 (r=0.40) deposition (both p<0.01). Multivariable analysis revealed that high glomerular IgM deposition remained an independent predictor of renal progression (adjusted HR=1.485, 95% CI 1.040 to 2.119, p=0.029).

**Conclusion:**

High glomerular IgM deposition emerged as an independent prognostic marker for adverse renal outcomes in LN, potentially outperforming other individual immune complexes. These findings highlight the pathogenic significance of IgM in LN and support its value in risk stratification and treatment guidance.

**WHAT IS ALREADY KNOWN ON THIS TOPIC**

- Glomerular immune complex deposition is a hallmark of LN, but the specific prognostic role of glomerular IgM deposition intensity has not been systematically studied. Prior studies typically treated IgM as part of the ‘full-house’ pattern without independent evaluation.

**WHAT THIS STUDY ADDS**

- This study identifies high glomerular IgM deposition as an independent predictor of poor renal outcomes in LN, distinct from other immune complex deposits. It demonstrates that patients with high IgM have more severe disease activity, complement activation and histopathological injury, even after adjusting for confounders.

**HOW THIS STUDY MIGHT AFFECT RESEARCH, PRACTICE OR POLICY**

- Glomerular IgM deposition scoring from routine immunofluorescence may serve as a simple, practical prognostic biomarker in LN. Recognising its association with aggressive disease could aid early risk stratification and inform personalised therapeutic strategies targeting high-risk LN patients.

## Introduction

 Lupus nephritis (LN), the most severe and frequent complication of SLE,^1 2^ significantly contributes to morbidity and mortality, primarily through accelerating progression to end-stage renal disease (ESRD). Despite advances in immunosuppressive therapies, a substantial proportion of patients with LN, estimated at 10–20%, still progress to ESRD within 5 years,^3^ underscoring the urgent need for more precise prognostic tools. Identifying reliable, easily accessible biomarkers that can predict renal outcomes is crucial for early risk stratification and tailoring therapeutic strategies in high-risk patients.

Histopathologically, LN is characterised by a distinctive ‘full-house’ immunofluorescence staining pattern—concurrent deposition of immunoglobulin G (IgG), immunoglobulin A (IgA), immunoglobulin M (IgM), complement component 1q (C1q) and complement 3 (C3).^4^ While previous studies have predominantly focused on IgG due to its established role in classical complement activation,^5 6^ emerging evidence suggests that this focus may overlook the potential contributions of other immunoglobulin subtypes. Recently, IgM, traditionally recognised for its role in antimicrobial defence, has garnered attention for its complex involvement in various forms of kidney injury,^7 8^ including diabetic nephropathy, focal segmental glomerulosclerosis^9^,^13^ and IgA nephropathy, in which glomerular IgM deposition often correlates with poorer outcomes. While transient IgM deposition might be involved in physiological clearance mechanisms, the persistent accumulation is increasingly implicated in promoting chronic inflammation and fibrosis.

Despite these insights from other nephropathies, the prognostic significance of glomerular IgM deposition in LN remains poorly defined. Most existing studies have treated IgM deposition as part of the ‘full-house’ pattern without a detailed quantitative assessment of its intensity or independent association with long-term renal prognosis.^14^ This gap in knowledge limits our understanding of IgM’s role and its potential as a distinct prognostic marker in LN.

Therefore, this study aims to systematically assess the prognostic significance of glomerular immune complex deposits in a large cohort of patients with biopsy-proven LN, with a specific focus on determining whether the intensity of glomerular IgM deposition is independently associated with adverse renal outcomes and all-cause mortality.

## Methods

### Study design and data collection

This retrospective cohort study evaluated the prognostic value of significant glomerular IgM deposition compared with other forms of immune complex deposits in LN patients. Patients diagnosed with LN via renal biopsy from January 1996 to December 2019 at the First Affiliated Hospital of Sun Yat-sen University were included. Eligible patients met both the 1997 American College of Rheumatology revised criteria^15^ for SLE and the 2003 International Society of Nephrology/Renal Pathology Society (ISN/RPS) classification criteria for LN.^16^ Exclusion criteria were: (1) ESRD at biopsy (n=219); (2) insufficient renal biopsy samples (fewer than 10 glomeruli) (n=148); (3) incomplete biopsy records (n=22); (4) concurrent malignancy (n=2) and (5) loss to follow-up (n=130). A total of 952 patients were ultimately analysed.

Baseline demographic and clinical characteristics, including assessments closest to renal biopsy and during follow-up, were obtained from the hospital’s standardised LN database (http://ln.medidata.cn). Anaemia was defined as haemoglobin <120 g/L in male and <110 g/L in female.^17^ Disease activity was assessed using the Systemic Lupus Erythematosus Disease Activity Index (SLEDAI).^18^ Renal function was evaluated by estimated glomerular filtration rate (eGFR) using the Chronic Kidney Disease Epidemiology Collaboration (CKD-EPI) equation.^19^

Treatment data were classified according to the initial induction regimen administered within 3 months of biopsy. For patients who switched regimens during follow-up, only the initial treatment was used for baseline group comparison. All patients received glucocorticoid therapy as the backbone of either induction or maintenance treatment. Immunosuppressive agents—including cyclophosphamide (CTX), mycophenolate mofetil (MMF) and calcineurin inhibitors (CNIs, such as tacrolimus or cyclosporine A)—were prescribed in combination with glucocorticoids, based on clinical indication, physician discretion and treatment guidelines during the study period. Patients who did not receive any of the above immunosuppressants were categorised into the other groups, which included those treated with glucocorticoid monotherapy, traditional Chinese medicine or other less commonly used immunomodulatory therapies.

### Renal histopathology

Renal biopsy specimens were evaluated using light microscopy, direct immunofluorescence and electron microscopy. Adequate biopsies were defined as at least 10 glomeruli for light microscopy and three glomeruli for immunofluorescence. LN was classified according to the ISN/RPS 2003 criteria,^16^ categorising patients into proliferative (classes III, IV and mixed V) or non-proliferative (classes I, II, pure V and class VI) groups.^20^ The diagnosis of TMA was made as described previously.^21^

Immunofluorescence staining intensity was graded semiquantitatively from negative (−) to strongly positive (++++).^20^ For each component, patients were assigned to a high group (≥++) or a low group (−/+), in accordance with previous studies that defined ≥++ as indicative of significant immune-complex deposition.^22^,^24^

Since the study includes archival data dating back to 1996, original pathology reports and available archival IF images were retrieved. These images, initially documented in contemporaneous diagnostic pathology reports at the time of renal biopsy, were re-evaluated independently by two experienced renal pathologists to ensure consistency across cases. When discrepancies were noted, consensus was reached through joint review to minimise interobserver variability.

### Study outcomes

The primary outcome was defined as either a doubling of serum creatinine from baseline or the development of ESRD, defined as eGFR ≤15 mL/min/1.73 m^2^, initiation of chronic dialysis or kidney transplantation. Secondary outcomes included all-cause mortality.

Patients were required to undergo regular comprehensive medical evaluations in person at our hospital or participate in telephone interviews conducted by experienced clinicians, at least twice per year. These evaluations were primarily for clinical management purposes and independent of this study. Follow-up continued until death, loss to follow-up or the study’s cut-off date of 30 September 2024.

### Statistical analysis

Data were analysed using SPSS (Version 25.0) and R (Version 4.1.3).^25^ Descriptive statistics were reported as mean±SD, median (IQR) or frequency (percentage), depending on distribution. Group comparisons were performed using independent-samples t tests, Mann-Whitney U tests or χ^2^ tests. Spearman’s correlation assessed associations between glomerular IgM deposition and other deposition markers, including IgA, IgG, C1q, C3 and fibrinogen.

Survival analyses employed Kaplan-Meier curves and log-rank tests. Cox proportional hazards models were used to identify risk factors for renal outcomes, with multivariate models adjusted for variables with p<0.1 in univariate analyses. Statistical significance was set at p<0.05. Sensitivity analyses addressed missing data and potential confounders. Data visualisation used R packages (gtools, nlme, survminer).

## Results

### Study population

A total of 952 patients were included, the majority of whom were female (82.4%), with a median age of 27 years at disease onset. The median follow-up duration was approximately 100 months, with a median interval of 3 months between symptom onset and renal biopsy. At baseline, median systolic and diastolic blood pressures were 124 mmHg and 80 mmHg, respectively. The median eGFR was 84.1 mL/min/1.73 m^2^, and median disease activity, assessed by the SLEDAI, was 16 (12–20) (table 1).

**Table 1:** Baseline characteristics among all 952 participants

Characteristic	All participants (n=952)
Age of onset (years)	27 (21–36)
Sex, male, n (%)	168 (17.6)
Time from onset to biopsy (month)	3 (1–13)
Systolic BP (mm Hg)	124 (113–139)
Diastolic BP (mm Hg)	80 (70–90)
Follow-up time (month)	100 (43–154)
SLEDAI	16 (12–20)
eGFR (mL/min/1.73 m^2^)	84.1 (50.5–120.3)
LN classification, n (%)	
I	9 (0.9)
II	86 (9.0)
III/III + V	150 (15.8)
IV/IV + V	538 (56.5)
V	161 (16.9)
VI	8 (0.8)
Intensity of glomerular IgA deposits, n (%)	
− to +	563 (59.1)
++ to ++++	389 (40.9)
Intensity of glomerular IgG deposits, n (%)	
− to +	258 (27.1)
++ to ++++	694 (72.9)
Intensity of glomerular IgM deposits, n (%)	
− to +	709 (74.5)
++ to ++++	243 (25.5)
Intensity of glomerular C3 deposits, n (%)	
− to +	317 (33.3)
++ to ++++	635 (66.7)
Intensity of glomerular C1q deposits, n (%)	
− to +	336 (35.3)
++ to ++++	616 (64.7)
Intensity of glomerular Fg deposits, n (%)	
− to +	846 (88.9)
++ to ++++	106 (11.1)
AI	7 (5–9)
CI	3 (2–4)
Electron microscopy	707 (74.3)
Treatment, n (%)	
Glucocorticoid+CTX, n (%)	324 (34.0)
Glucocorticoid+MMF, n (%)	179 (18.8)
Glucocorticoid+CNIs, n (%)	39 (4.1)
Others, n (%)	410 (43.1)

Numerical variables are reported as mean ± SD or median (IQR), while categorical variables are presented as counts (%).

AI, Activity Index; BP, blood pressure; C3, complement 3; CI, Chronicity Index; CKD-EPI, Chronic Kidney Disease Epidemiology Collaboration; CNIs, calcineurin inhibitors; C1q, complement component 1q; CTX, cyclophosphamide; eGFR, estimated glomerular filtration rate; Fg, fibrinogen; IgA, immunoglobulin A; IgG, immunoglobulin G; IgM, immunoglobulin M; LN, lupus nephritis; MMF, mycophenolate mofetil; SLEDAI, Systemic Lupus Erythematosus Disease Activity Index.

Regarding renal pathology, class IV/IV+V was the most common subtype, observed in 538 (56.5%) patients. Immunofluorescence analysis demonstrated variable intensities of immune complex deposition across patients. Notably, high-intensity IgM deposits were observed in approximately a quarter (25.5%) of the patients. In comparison, high-intensity deposits were more frequently observed for IgG (72.9%), followed by C3 (66.7%), C1q (64.7%) and, to a lesser extent, fibrinogen (11.1%). Electron microscopy data were available and complete for 707 (74.3%) patients, ensuring robust assessment of ultrastructural renal changes in a substantial subset of this study population.

In terms of treatment strategies, CTX was the most commonly used immunosuppressive agent, administered to 324 patients (34.0%), followed by MMF in 179 patients (18.8%) and CNIs in 39 patients (4.1%). Other treatment strategies were applied in 410 patients (43.1%).

### Association of glomerular immune complex deposits and renal and overall survival rate in patients with LN

**Figure 1:**
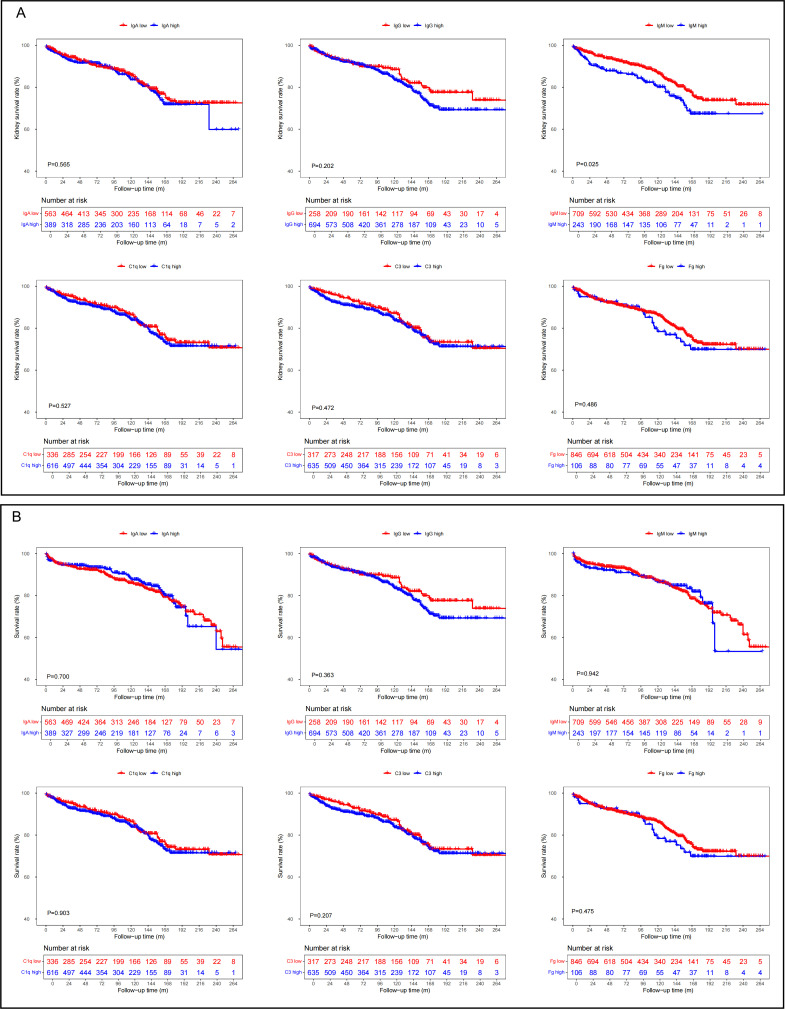
The association between glomerular immune complex deposits and renal and overall survival rates in patients with lupus nephritis. (A) The relationship between glomerular immune complex deposits and kidney outcome in patients with lupus nephritis. (B) The relationship between glomerular immune complex deposits and overall survival rates in patients with lupus nephritis. (low = − to +; high = ++ to ++++). C3, complement 3; Fg, fibrinogen; IgA, immunoglobulin A; IgG, immunoglobulin G; IgM, immunoglobulin M.

To evaluate the prognostic significance of immune complex deposits in LN, we conducted Kaplan-Meier survival analyses focusing on glomerular deposits of IgA, IgG, IgM, C3, C1q and fibrinogen. Patients were stratified into high and low deposition groups based on immunofluorescence intensity scores, as illustrated in figure 1.

Our analysis revealed that among all assessed immune complex components, only glomerular IgM deposition was significantly associated with renal survival. Specifically, patients with high glomerular IgM deposition exhibited a significantly worse renal survival compared with those with low glomerular IgM deposition (p=0.025) over a median follow-up period of 100 months (43–154 months). However, overall survival did not significantly differ between IgM deposition groups (p=0.94).

Similar analyses for IgA, IgG, C3, C1q and fibrinogen deposits showed no statistically significant association with renal or overall survival (all p>0.05).

### Comparison of demographic, clinical and treatment parameters between low and high glomerular IgM deposition groups

Patients were categorised based on glomerular IgM deposition intensity observed during biopsy. The glomerular IgM low deposition group (− to +) included 709 patients (74.5%), while the high glomerular IgM deposition group (++ to ++++) comprised 243 patients (25.5%). Demographic and clinical characteristics of these groups are summarised in table 2.

**Table 2:** Clinical, demographic and treatment characteristics of lupus nephritis patients with low and high glomerular IgM deposition

Characteristic	Glomerular IgM low (n=709)	Glomerular IgM high (n=243)	P value
Age of onset (years)	26 (19–35)	26 (20–34)	0.794
Time from onset to biopsy (month)	3 (1–14)	3 (1–13)	0.92
Sex, male, n (%)	135 (19.0)	33 (13.6)	0.054
Hypertension, n (%)	229 (32.3)	94 (38.7)	0.07
Systolic BP (mm Hg)	122 (111–137)	127 (115–140)	0.006
Diastolic BP (mm Hg)	80 (70.0–89.5)	82 (74.0–91.0)	0.018
Fever, n (%)	195 (27.5)	63 (25.9)	0.633
Rash, n (%)	214 (30.2)	79 (32.5)	0.498
Photosensitivity, n (%)	64 (9.0)	26 (10.7)	0.442
Mucosal ulcer, n (%)	43 (6.1)	6 (2.5)	0.029
Raynaud’s phenomenon, n (%)	9 (1.3)	2 (0.8)	0.574
Alopecia, n (%)	99 (14.0)	47 (19.3)	0.045
Oedema, n (%)	497 (70.1)	59 (75.7)	0.094
Anaemia, n (%)	466 (65.7)	171 (70.4)	0.184
SLEDAI Score	15 (12–18)	16 (12–20)	<0.001
Serum albumin (g/L)	26.7 (21.0–32.0)	25 (20.0–30.4)	0.004
eGFR (mL/min/1.73 m^2^)	82 (49.2–120.9)	89.1 (52.8–119.8)	0.86
Blood urea nitrogen (mmol/L)	7.2 (4.8–11.8)	7.2 (5.0–11.1)	0.715
Serum creatinine (μmol/L)	82 (58.0–123.5)	79 (58.0–131.0)	0.818
Uric acid (μmol/L)	419 (321–501)	401 (319–505)	0.601
Proteinuria (g/24 hours)	2.1 (0.9–4.2)	2.2 (1.2–4.1)	0.335
HDL-C (mmol/L)	1.1 (0.8–1.4)	1 (0.7–1.3)	0.005
ApoA (g/L)	1.2 (1.0–1.5)	1.1 (0.9–1.3)	0.011
Positive ANA, n (%)	684 (96.5)	240 (98.8)	0.068
Positive anti-dsDNA, n (%)	582 (82.1)	213 (87.7)	0.044
Positive anti-SSA, n (%)	391 (55.1)	148 (60.9)	0.118
Positive anti-SSB, n (%)	131 (18.5)	53 (21.8)	0.256
Positive anticardiolipin IgM, n (%)	112 (15.8)	43 (17.7)	0.489
Positive anticardiolipin IgG, n (%)	144 (20.3)	56 (23.0)	0.366
Positive anti-Sm, n (%)	150 (21.2)	76 (31.3)	0.001
Positive anti-RNP, n (%)	238 (33.6)	95 (39.1)	0.119
Serum IgG (g/L)	10.8 (7.0–15.2)	11.8 (7.3–17.5)	0.178
Serum IgA (g/L)	2.2 (1.6–2.9)	2.3 (1.6–2.9)	0.656
Serum IgM (g/L)	1.0 (0.6–1.4)	1.2 (0.7–1.6)	0.002
C3 (g/L)	0.5 (0.3–0.6)	0.4 (0.3–0.6)	0.013
C4 (g/L)	0.10 (0.07–0.18)	0.08 (0.06–0.15)	<0.001
Treatment, n (%)			0.723
Glucocorticoid+CTX, n (%)	243 (34.3)	81 (33.3)	
Glucocorticoid+MMF, n (%)	126 (17.8)	53 (21.8)	
Glucocorticoid+CNIs, n (%)	30 (4.2)	9 (3.7)	
Others, n (%)	310 (43.7)	100 (42.2)	

Numerical variables are reported as mean ± SD or median (IQR), while categorical variables are presented as counts (%).

P value was adjusted for multiple comparisons.

anti-RNP, anti-ribonucleoprotein antibody; anti-Sm, anti-Smith antibody; anti-SSA, anti-Sjögren’s-syndrome-related antigen A; anti-SSB, anti-Sjögren’s-syndrome-related antigen B; ApoA, apolipoprotein A; BP, blood pressure; C3, complement 3; C4, complement 4; CNIs, calcineurin inhibitors; CTX, cyclophosphamide; eGFR, estimated glomerular filtration rate; HDL-C, high-density lipoprotein cholesterol; IgA, immunoglobulin A; IgG, immunoglobulin G; IgM, immunoglobulin M; MMF, mycophenolate mofetil; SLEDAI, Systemic Lupus Erythematosus Disease Activity Index.

The median age at onset and the median duration from disease onset to biopsy were similar between the two groups, both at 26 years and 3 months, respectively, suggesting comparable disease progression timeline. Although the proportion of male patients was slightly higher in the low glomerular IgM deposition group (19% vs 13.6%), this difference was not statistically significant (p=0.054). While hypertension prevalence appeared higher in the high glomerular IgM deposition group (38.7% vs 32.3%), the difference did not reach statistical significance (p=0.07). However, significantly elevated systolic and diastolic pressures were observed in patients with high glomerular IgM deposition (127 mmHg vs 122 mmHg, p=0.006; 82 mmHg vs 80mmHg, p=0.018).

The prevalence of most clinical manifestations—including fever, rash, photosensitivity and Raynaud’s phenomenon—did not differ significantly between groups. However, patients in the high glomerular IgM deposition group exhibited a significantly lower incidence of mucosal ulcers (p=0.029) and a higher incidence of alopecia (19.3% vs 14%, p=0.045). Additionally, the SLEDAI scores were significantly higher in the high glomerular IgM deposition group (16 (12–20) vs 15 (12–18), p<0.001).

Laboratory analyses revealed notable differences between groups, with patients in the high glomerular IgM deposition group deposition showing significantly lower serum albumin levels (25 (20–30.4) g/L vs 26.7 (21–32) g/L, p=0.004) and higher serum IgM concentrations (1.2 (0.7–1.6) g/L vs 1 (0.6–1.4) g/L, p=0.002). No significant differences were observed between groups regarding anaemia, eGFR, blood urea nitrogen, serum creatinine, uric acid or 24-hour proteinuria. Additionally, patients in the high glomerular IgM deposition group exhibited significantly lower high-density lipoprotein cholesterol levels (1.0 (0.7–1.3) mmol/L vs 1.1 (0.8–1.4) mmol/L, p=0.005) and apolipoprotein A levels (1.1 (0.9–1.3) g/L vs 1.2 (1–1.5) g/L, p=0.011), reflecting a potentially unfavourable lipid profile.

Autoantibody analyses showed significantly higher proportions of positive anti-dsDNA (87.7% vs 82.1%, p=0.044) and anti-Smith (anti-Sm antibodies (31.3% vs 21.2%, p=0.001) in the high glomerular deposition. Conversely, no significant differences were observed in the positivity rates of ANA (98.8% vs 96.5%, p=0.068), anti-Sjögren’s-syndrome-related antigen A (anti-SSA) (60.9% vs 55.1%, p=0.118), anti-Sjögren’s-syndrome-related antigen B (anti-SSB) (21.8% vs 18.5%, p=0.256) or anti-ribonucleoprotein antibody (anti-RNP) (39.1% vs 33.6%, p=0.119). Similarly, the prevalence of anticardiolipin antibodies, both IgM (17.7% vs 15.8%, p=0.489) and IgG (23.0% vs 20.3%, p=0.366), did not differ significantly between groups.

Complement component levels (C3 and C4) were significantly lower in patients with high glomerular IgM deposition compared with the low deposition group, with median levels of 0.4 (0.3–0.6) g/L vs 0.5 (0.3–0.6) g/L for C3 (p=0.013) and 0.08 (0.06–0.15) g/L vs 0.1 (0.07–0.18) g/L for C4 (p<0.001), respectively.

In terms of treatment strategies, both groups exhibited comparable therapeutic patterns. The proportion of patients receiving CTX was similar between the high and low glomerular IgM deposition groups (33.3% vs 34.3%), as was the use of MMF (21.8% vs 17.8%) and CNIs (3.7% vs 4.2%). The proportion of patients treated with other regimens was also comparable (42.2% vs 43.7%). Overall, treatment distributions were well balanced between the two groups, with no statistically significant difference (p=0.723).

### Comparison of pathologic parameters between low and high glomerular IgM deposition groups

Table 3 illustrates substantial differences between the low and high glomerular IgM groups in key pathological features of LN. Specifically, the high glomerular IgM deposition group exhibited a significantly higher prevalence of proliferative LN (82.3% vs 68.4%; p<0.001).

**Table 3:** The pathological parameters of lupus nephritis patients with low and high glomerular IgM deposition

Characteristic	Glomerular IgM low (n=709)	Glomerular IgM high (n=243)	P value
LN classification, n (%)			<0.001
I/II/V/VI	224 (31.6)	43 (17.7)	
III/IV/III+V/IV+V	485 (68.4)	200 (82.3)	
Glomerular leucocyte infiltration, n (%)			0.001
None	331 (46.7)	92 (37.9)	
<25%	203 (28.6)	56 (23.0)	
25–50%	137 (19.3)	82 (33.7)	
>50%	38 (5.4)	13 (5.3)	
Endocapillary hypercellularity, n (%)			<0.001
None	315 (44.4)	75 (30.9)	
Focal segmental	286 (40.3)	109 (44.9)	
Diffuse	108 (15.2)	59 (24.3)	
Crescents	0 (0–4)	1 (0–4)	0.29
Platinum loop, n (%)	160 (22.6)	91 (37.4)	<0.001
Microthrombus, n (%)	101 (14.2)	68 (27.9)	<0.001
TMA, n (%)	21 (3.0)	6 (2.5)	0.69
Karyorrhexis, n (%)			0.006
None	513 (72.4)	155 (63.8)	
<25%	151 (21.3)	59 (24.3)	
25–50%	37 (5.2)	25 (10.3)	
>50%	8 (1.1)	4 (1.6)	
AI	7 (5-9)	7 (6-10)	<0.001
CI	3 (2-4)	3 (2-4)	0.008
Interstitial inflammation, n (%)			0.76
None	223 (31.5)	68 (28.0)	
<25%	338 (47.7)	136 (56.0)	
25–50%	93 (13.1)	32 (13.2)	
50–75%	23 (3.2)	3 (1.2)	
>75%	32 (4.5)	4 (1.6)	
Interstitial fibrosis, n (%)			0.597
None	405 (57.1)	143 (58.8)	
<25%	226 (31.9)	76 (31.3)	
25–50%	66 (9.3)	20 (8.2)	
50–75%	8 (1.1)	2 (0.8)	
>75%	4 (0.6)	2 (0.8)	
Tubular atrophy, n (%)			0.222
None	284 (40.1)	109 (44.9)	
<25%	286 (40.3)	90 (37.0)	
25–50%	91 (12.8)	20 (12.3)	
50–75%	27 (3.8)	9 (3.7)	
>75%	21 (3.0)	5 (2.1)	
Renal tubular necrosis, n (%)	25 (3.5)	12 (4.9)	0.326
Renal interstitial oedema, n (%)	133 (18.8)	71 (29.2)	<0.001
Mesangial cell and matrix hyperplasia, n (%)			0.028
None	30 (4.2)	11 (4.3)	
<25%	233 (32.9)	53 (21.8)	
25–50%	237 (33.4)	100 (41.2)	
>50%	209 (29.5)	79 (32.5)	
IgA immunofluorescence intensity, n (%)			<0.001
− to +	502 (70.8)	61 (25.1)	
++ to ++++	207 (29.2)	182 (74.9)	
IgG immunofluorescence intensity, n (%)			<0.001
− to +	233 (32.9)	25 (10.3)	
++ to ++++	476 (67.1)	218 (89.7)	
C1q immunofluorescence intensity, n (%)			<0.001
− to +	293 (41.3)	43 (17.7)	
++ to ++++	416 (58.7)	200 (82.3)	
C3 immunofluorescence intensity, n (%)			<0.001
− to +	278 (39.2)	39 (16.0)	
++ to ++++	431 (60.8)	204 (84.0)	
Fg immunofluorescence intensity, n (%)			<0.001
− to +	667 (94.1)	179 (73.7)	
++ to ++++	42 (5.9)	64 (26.3)	
Electron microscopy			
Subepithelial deposits, n (%)	333/518 (64.3)	128/189 (67.7)	0.396
Basement membrane deposits, n (%)	86/518 (16.6)	30/189 (15.9)	0.809
Subendothelial deposits, n (%)	193/518 (37.3)	113/189 (59.8)	<0.001
Mesangial/paramesangial deposits, n (%)	39/518 (7.5)	26/189 (13.8)	0.011
Foot process effacement, n (%)			0.009
None	27/518 (5.2)	3/189 (1.6)	
Partial fusion	104/518 (20.1)	28/189 (14.8)	
Diffuse fusion	387/518 (74.7)	158/189 (83.6)	

Numerical variables are reported as mean ± SD or median (IQR), while categorical variables are presented as counts (%).

AI, Activity Index; C3, complement 3; CI, Chronicity Index; C1q, complement component 1q; Fg, fibrinogen; IgA, immunoglobulin A; IgG, immunoglobulin G; IgM, immunoglobulin M; LN, lupus nephritis; TMA, thrombotic microangiopathy.

Compared with the low glomerular IgM deposition group, the high glomerular IgM deposition group exhibited a higher incidence of these severe pathological findings. Endocapillary hypercellularity, glomerular leucocyte infiltration, karyorrhexis, platinum loop, microthrombus and renal interstitial oedema also demonstrated marked disparities (p<0.001, p=0.001, p=0.006, p<0.001, p<0.001, p<0.001). Among patients with low glomerular IgM deposition, 21 (3.0%) had TMA, whereas 6 (2.5%) were observed in the high glomerular IgM deposition group. No significant difference was found between the groups (p=0.69). Regarding tubulointerstitial lesions, no significant differences were observed in interstitial inflammation (p=0.76), interstitial fibrosis (p=0.597) or tubular atrophy (p=0.222).

Furthermore, the median Activity Index Score was significantly elevated in the high glomerular IgM deposition group (7 (6–10)) compared with the low glomerular IgM group (7 (5–9)) (p<0.001).

Significant differences in immunofluorescence intensity were observed for IgA, IgG, C1q, C3 and fibrinogen (all p<0.001). Patients with glomerular IgM elevation displayed stronger immunofluorescence staining for IgG, C1q, C3 and fibrinogen, suggesting a correlation between increased glomerular IgM and intensified immune complex deposition.

Additionally, electron microscopy findings (table 3) provided further insights into the characteristics of these patient groups. Of the 952 LN patients, 707 (74.3%) had valid electron microscopy results. The high glomerular IgM deposition group exhibited significantly higher frequencies of deposits, particularly subendothelial (59.8%, p<0.001) and mesangial/paramesangial deposits (13.8%, p=0.011). Moreover, 28 patients (14.8%) in this group showed partial foot process fusion, while 158 (83.6%) exhibited diffuse foot process fusion. Importantly, the severity of foot process fusion was notably greater in the high glomerular IgM deposition group compared with the low glomerular IgM deposition group (p=0.009).

### Glomerular IgM deposition and correlations with other glomerular immunoglobulins and complement deposition in patients with LN

**Figure 2:**
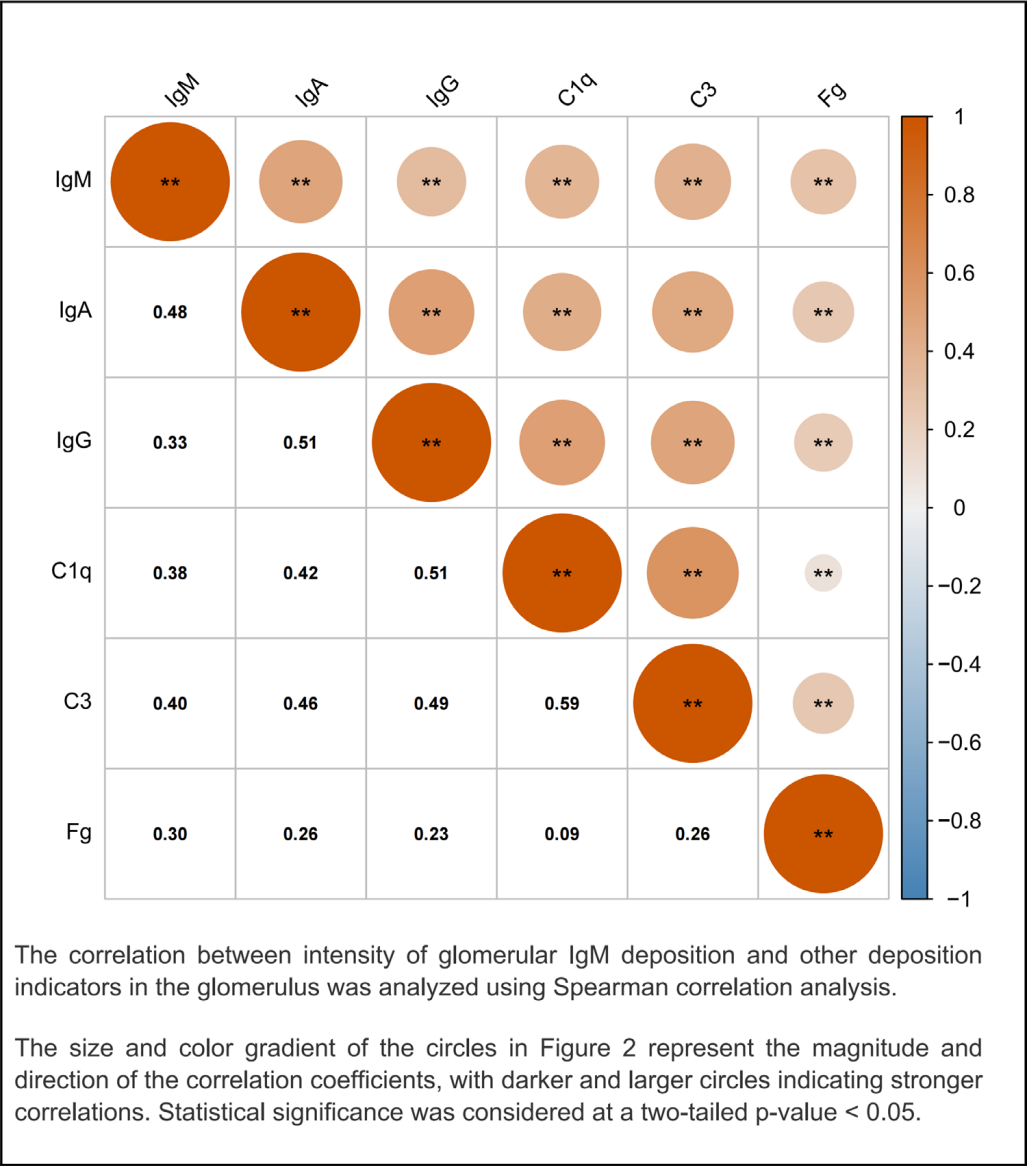
Glomerular IgM deposition and correlations with other glomerular deposition in patients with lupus nephritis. The correlation between intensity of glomerular IgM deposition and other deposition indicators in the glomerulus was analysed using Spearman correlation analysis. The size and colour gradient of the circles represent the magnitude and direction of the correlation coefficients, with darker and larger circles indicating stronger correlations. Statistical significance was considered at a two-tailed p<0.05. C3, complement 3; C1q, complement component 1q; Fg, fibrinogen; IgA, immunoglobulin A; IgG, immunoglobulin G; IgM, immunoglobulin M.

To investigate the clinical significance of glomerular IgM deposition in LN, we explored its relationship with other key glomerular deposition markers. As shown in figure 2, a Spearman analysis revealed significant positive correlations between IgM and several other immune components, including IgG (r=0.33, p<0.001), IgA (r=0.48, p<0.001), C3 (r=0.40, p<0.001) and C1q (r=0.38, p<0.001). Notably, the correlations between IgM and IgA, as well as IgM and C3, were among the strongest, underscoring the potentially important role of IgM in immune complex formation in LN.

### Glomerular IgM deposition independently contributes to poor kidney prognosis in patients with LN

To assess whether glomerular IgM deposition independently predicts renal prognosis in LN, we used a Cox proportional hazards model to assess the risk factors for renal outcomes and overall survival in LN patients with high IgM deposits.

**Figure 3:**
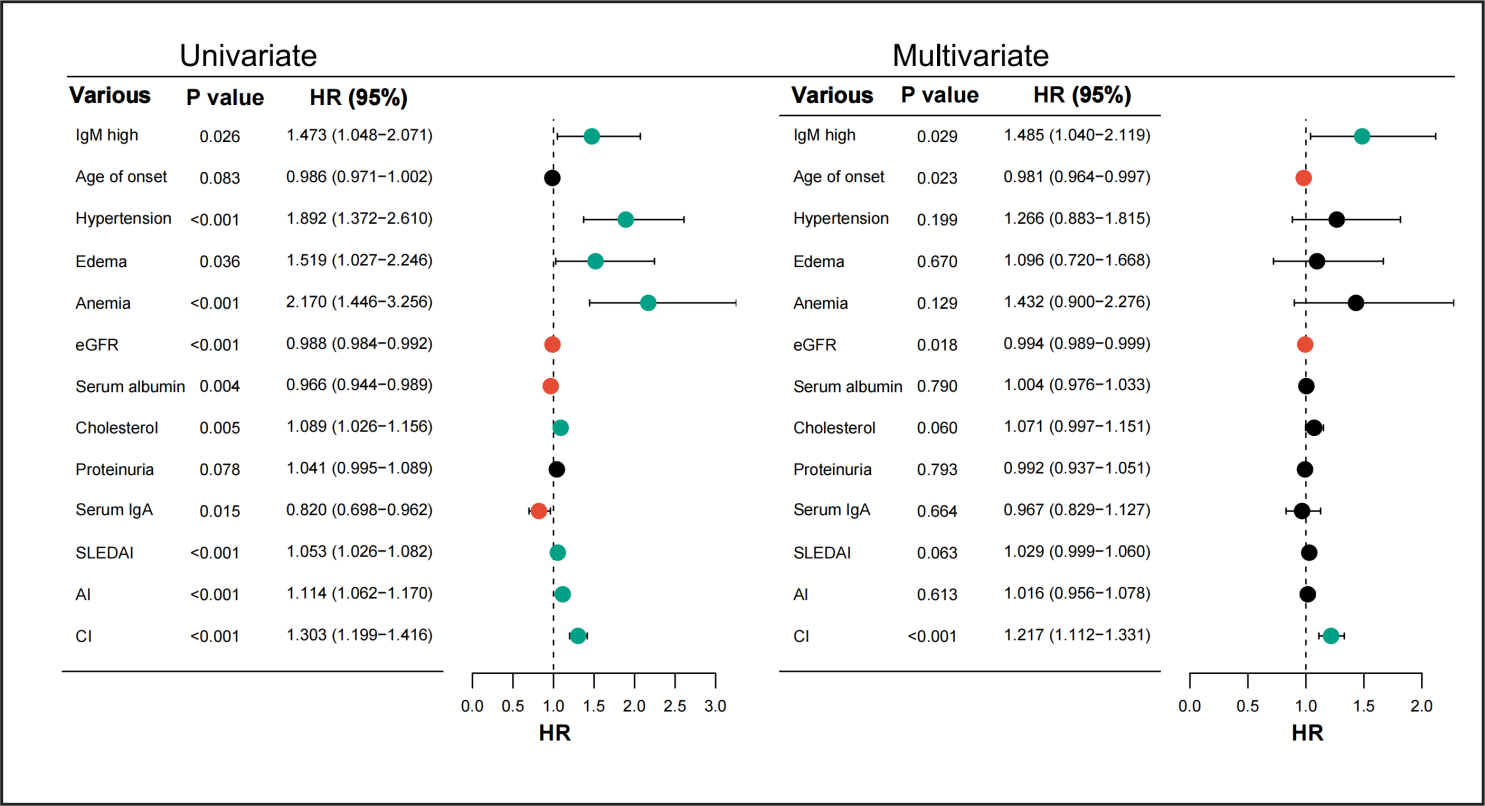
Risk factors for poor kidney prognosis in patients with lupus nephritis. AI, Activity Index; CI, Chronicity Index; eGFR, estimated glomerular filtration rate; IgA, immunoglobulin A; IgM, immunoglobulin M; SLEDAI, Systemic Lupus Erythematosus Disease Activity Index.

As shown in figure 3, patients with higher levels of glomerular IgM deposition demonstrated significantly worse renal outcomes. In the univariate analysis, high-intensity glomerular IgM deposition was a significant risk factor for adverse kidney outcomes, with a HR of 1.473 (95% CI, 1.048 to 2.071; p=0.026). This association remained statistically significant in the multivariate analysis, with an adjusted HR of 1.485 (95% CI, 1.040 to 2.119; p=0.029), confirming the independent role of IgM in poor renal outcomes.

In addition to IgM deposition, other factors were found to influence renal outcomes in the multivariate model. These included age of onset (HR, 0.981; 95% CI, 0.964 to 0.997; p=0.023), eGFR (HR, 0.994; 95% CI, 0.989 to 0.999; p=0.018) and the Chronicity Index (HR, 1.217; 95% CI, 1.112 to 1.331; p<0.001). Variance inflation factor (VIF) analysis confirmed no significant multicollinearity among the included covariates, with all VIFs below 2.0 (online supplemental figure S4). These findings highlight the multifaceted nature of renal prognosis in LN, with glomerular IgM deposition serving as an independent and significant contributor to poor kidney outcomes.

### Subgroup analyses reveal the prognostic relevance of IgM deposition beyond complement codeposition

To further explore whether the prognostic value of glomerular IgM deposition in LN is merely a reflection of concurrent complement activation, we conducted stratified survival analyses based on the levels of C3/C1q deposition.

As shown in online supplemental figure S5, among patients with high glomerular IgM deposition, the extent of glomerular C3/C1q deposition did not significantly affect either renal survival (p=0.624) or overall survival (p=0.363).

Conversely, online supplemental figure S6 depicts survival outcomes among patients with high C3/C1q deposition, stratified by IgM levels. In this subgroup, patients with high glomerular IgM deposition showed a clear trend towards poorer renal survival compared with those with low glomerular IgM deposition (p=0.051), although no significant difference was observed in overall survival (p=0.971).

Furthermore, as shown in online supplemental figure S7, we examined patients with low C3/C1q deposition. In this group, high glomerular IgM deposition was associated with a trend towards worse renal survival (p=0.070), while overall survival remained comparable between groups (p=0.982).

## Discussion

IgM, the first antibody produced during a humoral immune response, is known for its ability to form polymers, with the pentameric form adopting an asymmetrical structure.^26^ However, its deposition in the glomerulus may contribute to renal injury.^27^ This retrospective cohort study demonstrated that high-intensity glomerular IgM deposition is an independent predictor of adverse renal outcomes in LN, distinguishing itself from other components of the classical ‘full-house’ immunofluorescence pattern such as IgG, IgA, C1q and C3. This key finding supports glomerular IgM as a distinct and clinically relevant prognostic marker, warranting greater attention in routine pathological assessment and risk stratification.

Our findings contribute to a nuanced understanding of immune complex-mediated injury in LN. While the ‘full-house’ pattern is a diagnostic hallmark,^4^ a detailed dissection of the prognostic weight of each component has been less clear. Many studies have historically focused on IgG due to its established role in classical complement activation and immune-mediated injury.^28 29^ Although some previous investigations have noted the presence of IgM in LN or included it within the ‘full-house’ pattern, most did not quantitatively assess its deposition intensity.^30^ By leveraging a substantial cohort and long-term follow-up, our study quantitatively assessed IgM deposition and demonstrated its strong association with adverse renal outcomes. This observation aligns with emerging evidence suggesting that IgM deposition is associated with more severe disease and poorer outcomes.^9^,^13^

In our study, patients with high glomerular IgM deposition not only exhibited significantly worse renal outcomes but also demonstrated higher disease activity and more aggressive histopathological features. The high glomerular IgM deposition group exhibited higher SLEDAI scores, elevated blood pressure and more severe lesions, collectively indicating a more aggressive LN phenotype.^31 32^ Notably, despite similar baseline 24-hour proteinuria, patients with high glomerular IgM deposition exhibited lower serum albumin and more severe foot process effacement, potentially indicating a more injurious form of proteinuria or a longer subclinical phase leading to greater cumulative damage.^33 34^

Several interconnected mechanisms may underpin the detrimental role of glomerular IgM deposition in LN. First, IgM is a potent activator of the classical complement pathway.^35^ The observed lower serum C3 and C4 levels in the high glomerular IgM deposition group suggest increased complement consumption, and IgM-triggered activation may further amplify glomerular inflammation and injury.^27 36 37^ Second, the large pentameric structure of IgM may promote immune complex trapping and hinder clearance,^38 39^ potentially contributing to endothelial damage and microthrombosis—both more frequent in the high glomerular IgM deposition group.^40^ Thirdly, natural IgM can bind to neoepitopes or recognise damage-associated molecular patterns exposed on damaged tissues or apoptotic cells,^36^ particularly common in lupus flares,^41^ potentially perpetuating a cycle of inflammation and injury.^42^

Experimental evidence further supports a pathogenic role for IgM. For instance, Ito *et al*^7^ showed that glomerular IgM deposition can exacerbate disease by promoting macrophage infiltration and enhancing lesion severity in murine lupus models. Additionally, IgM may contribute to vascular alterations through receptors such as FcμR and pIgR.^43^ The above findings are consistent with existing literature, which identifies glomerular IgM deposition as a major contributor to complement activation and tissue injury in glomerulonephritis,^30 44^ although this requires more specific investigation in the context of LN.

Although IgM was frequently codeposited with complement components such as C3 and C1q, subgroup analyses suggest that its prognostic impact may not be solely attributable to broader immune complex burden. Notably, among patients with high complement deposition, those with high IgM still exhibited worse renal outcomes, supporting an independent contribution of IgM to disease progression.

The clinical implications of our findings are substantial. Glomerular IgM deposition intensity is a readily assessable parameter from routine renal biopsy immunofluorescence. Our results advocate for its elevation to a key prognostic indicator in LN. Patients with high glomerular IgM deposition may benefit from closer monitoring or tailored treatment strategies, though prospective studies are needed to validate its predictive utility in therapeutic decision-making.

This study possesses several strengths, including its relatively large sample size, long duration of follow-up and comprehensive collection of clinical and histopathological data. Nevertheless, certain limitations must be acknowledged. Its retrospective, single-centre design may introduce selection bias and limit generalisability. We acknowledge potential variability in histological processing and interpretation over the 23-year study period, despite re-evaluation by experienced pathologists to ensure consistency. However, subtle variations cannot be entirely excluded. While we adjusted for multiple confounders, residual confounding is possible. In addition, treatment data were obtained from the standardised LN database and classified according to the initial induction regimen within 3 months after biopsy. For patients who changed regimens during follow-up, only the initial treatment was used for baseline comparison. Finally, although multivariate Cox regression and VIF analyses were performed to address confounding and multicollinearity, causal inference remains limited. Our findings underscore a robust association between glomerular IgM and renal outcomes, yet further mechanistic and interventional studies are warranted to clarify its role in LN.

## Conclusions

In conclusion, this study identifies high-intensity glomerular IgM deposition, distinct from other immune complex deposits, as a significant and independent predictor of adverse renal outcomes in a large cohort of LN patients. Associated with greater disease activity, more severe renal pathology and glomerular IgM deposition intensity holds promise as a clinically accessible biomarker for risk stratification, potentially guiding more personalised management of LN.

## Supplementary material

10.1136/lupus-2025-001708 online supplemental file 1

10.1136/lupus-2025-001708 online supplemental file 2

## Data Availability

Data are available upon reasonable request.
