# Supplementary material for: Significant glomerular IgM deposition predicts poorer kidney outcomes in lupus nephritis compared with other forms of immune complex deposits
Source: Lupus Sci Med. 2025 Oct 10;12(2):e001708. doi: 10.1136/lupus-2025-001708 (PMC12516977; doi:10.1136/lupus-2025-001708)
Supplement: online supplemental file 1 [file lupus-12-2-s001.pdf]

Significant Glomerular IgM deposition predicts poorer kidney outcomes in Lupus nephritis Compared to Other Forms of Immune Complex Deposits

Methods and cohort

The First Affiliated Hospital of Sun Yat-sen University, 1996-2019

LN patients (n=952)

Median follow-up time 100 months

Doubling of serum creatinine or End-stage renal disease (ESRD)

Results

The intensity of glomerular immune complex deposition

IgA IgG IgM C1q C3 Fg
(- to + VS. ++ to++++)

High IgM group (n=243)
(++ to ++++)

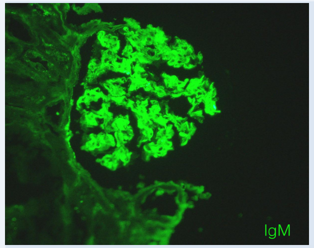

High glomerular IgM deposition is associated with poorer renal outcomes in LN patients

hazard ratio (95% confidence interval)	**1.485 (1.040-2.119)**

Blood pressure ↑

SLE disease activity index ↑

Proliferative LN ↑

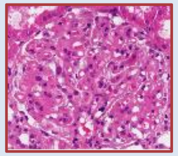

Serum complement 3/4 ↓

C3 C4

Conclusion High glomerular IgM deposition in lupus nephritis indicates more active disease and predicts worse renal outcomes than other immune deposits.
