# Supplementary material for: Significant glomerular IgM deposition predicts poorer kidney outcomes in lupus nephritis compared with other forms of immune complex deposits
Source: Lupus Sci Med. 2025 Oct 10;12(2):e001708. doi: 10.1136/lupus-2025-001708 (PMC12516977; doi:10.1136/lupus-2025-001708)

Figure S1. Patient Enrollment and Longitudinal Follow-up.

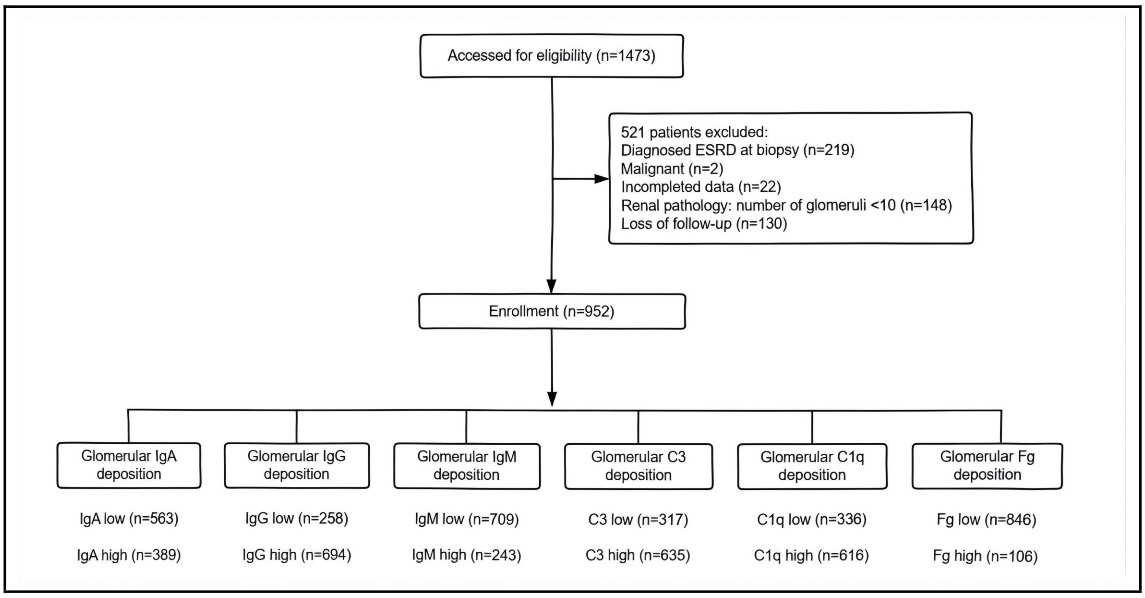

Figure S2. Glomerular IgM deposition and associated ultrastructural changes in lupus nephritis.

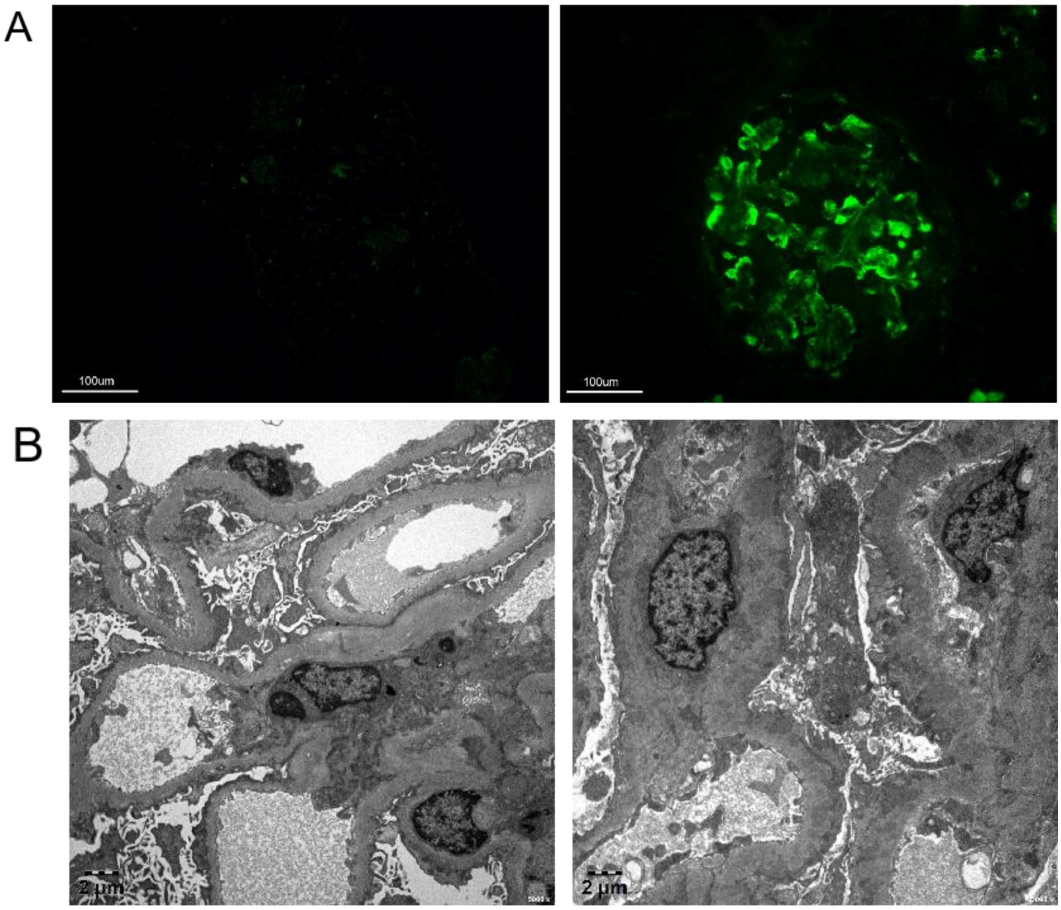

A: Representative immunofluorescence staining of IgM in renal biopsies. Green fluorescence indicates glomerular IgM deposition. IgM-low group (left) shows weak signal with minimal deposition; IgM-high group (right) shows intense, clustered IgM staining along capillary loops. Scale bar = 100 μm.

B: Transmission electron microscopy images. IgM-low group (left) shows relatively preserved glomerular structure with mild podocyte foot process effacement. IgM-high group (right) reveals marked ultrastructural damage, including extensive foot process fusion, endothelial swelling, and mesangial expansion. Scale bar = 2 μm.

Figure S3. The association between glomerular IgM deposits and renal and overall survival rates in patients with lupus nephritis.

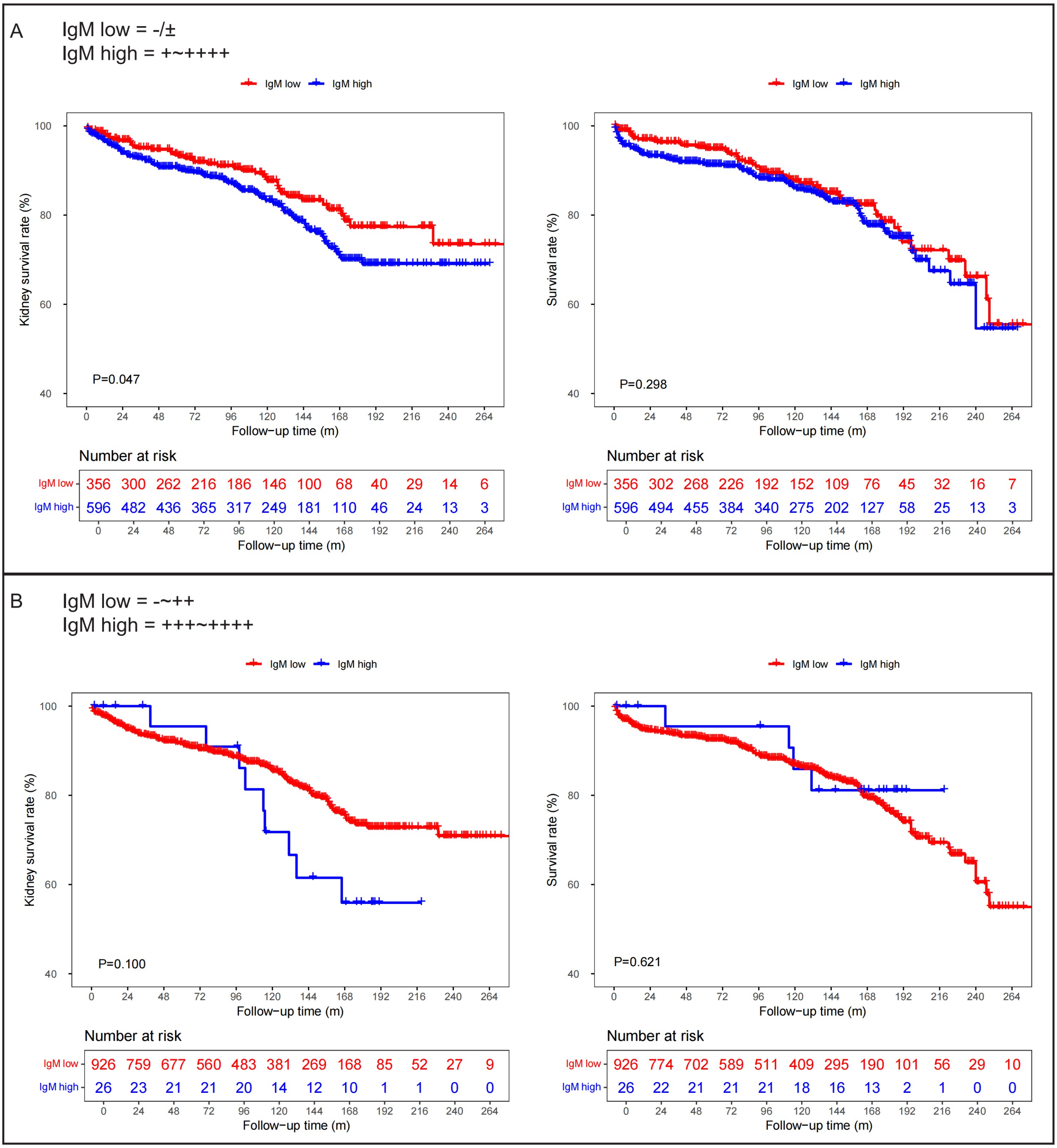

Figure S4. Variance inflation factors (VIFs) analysis of covariates in the multivariate Cox model.

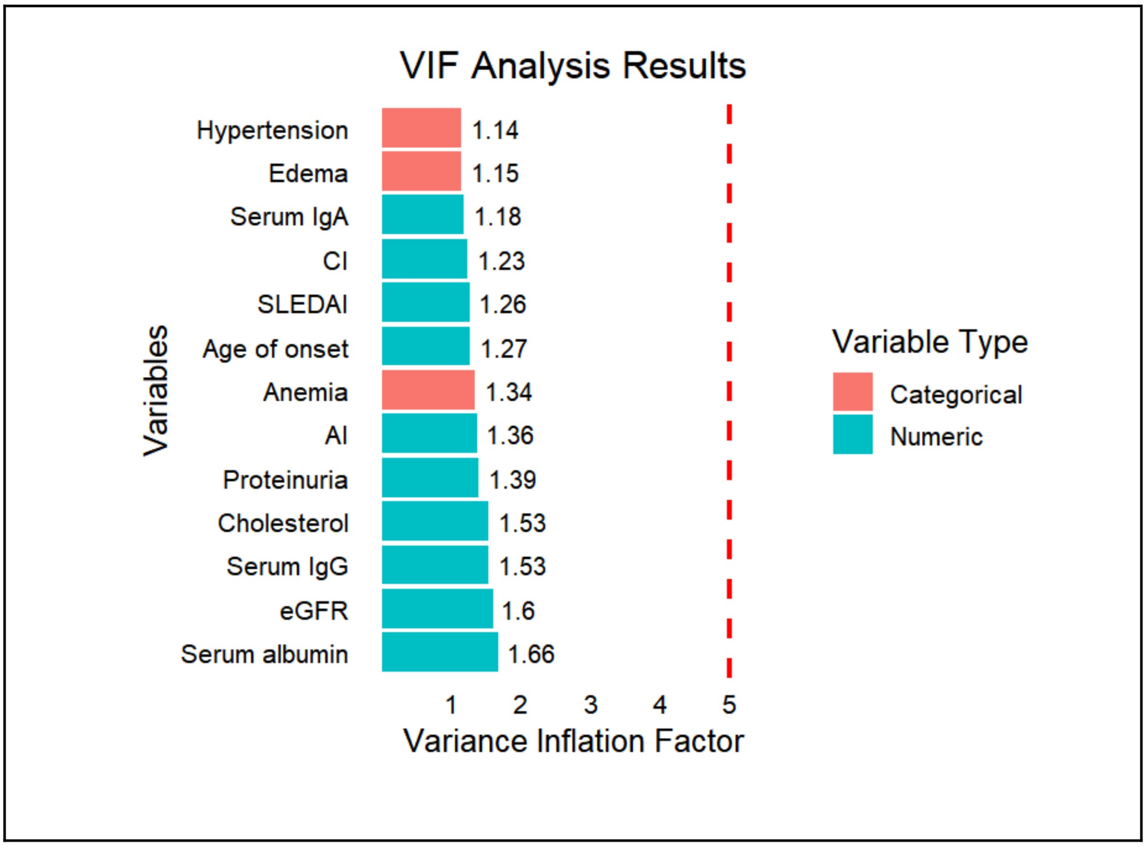

Figure S5. Prognostic impact of glomerular C3/C1q deposition on renal and overall survival in lupus nephritis patients with high IgM deposition.

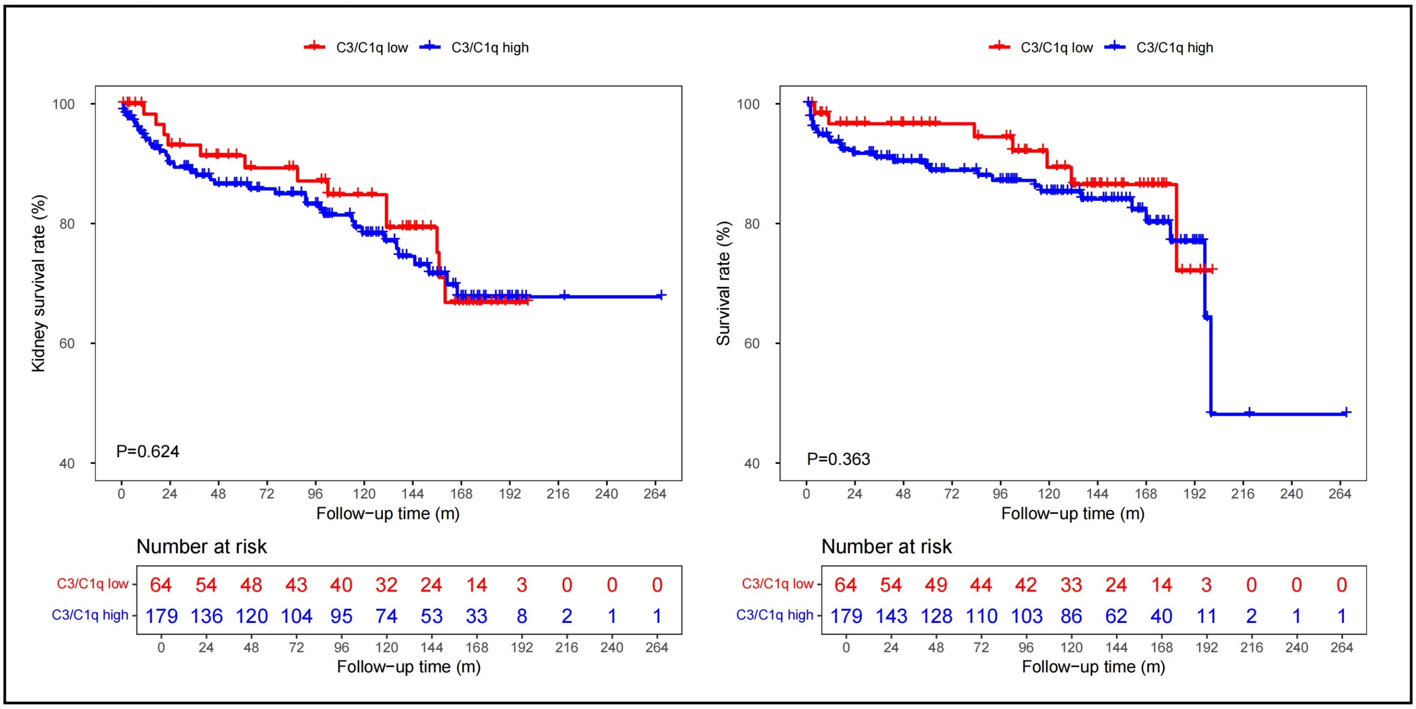

(C3/C1q low=− to +; C3/C1q high = ++ to ++++)

Figure S6. Prognostic impact of glomerular IgM deposition on renal and overall survival in lupus nephritis patients with high C3/C1q deposition.

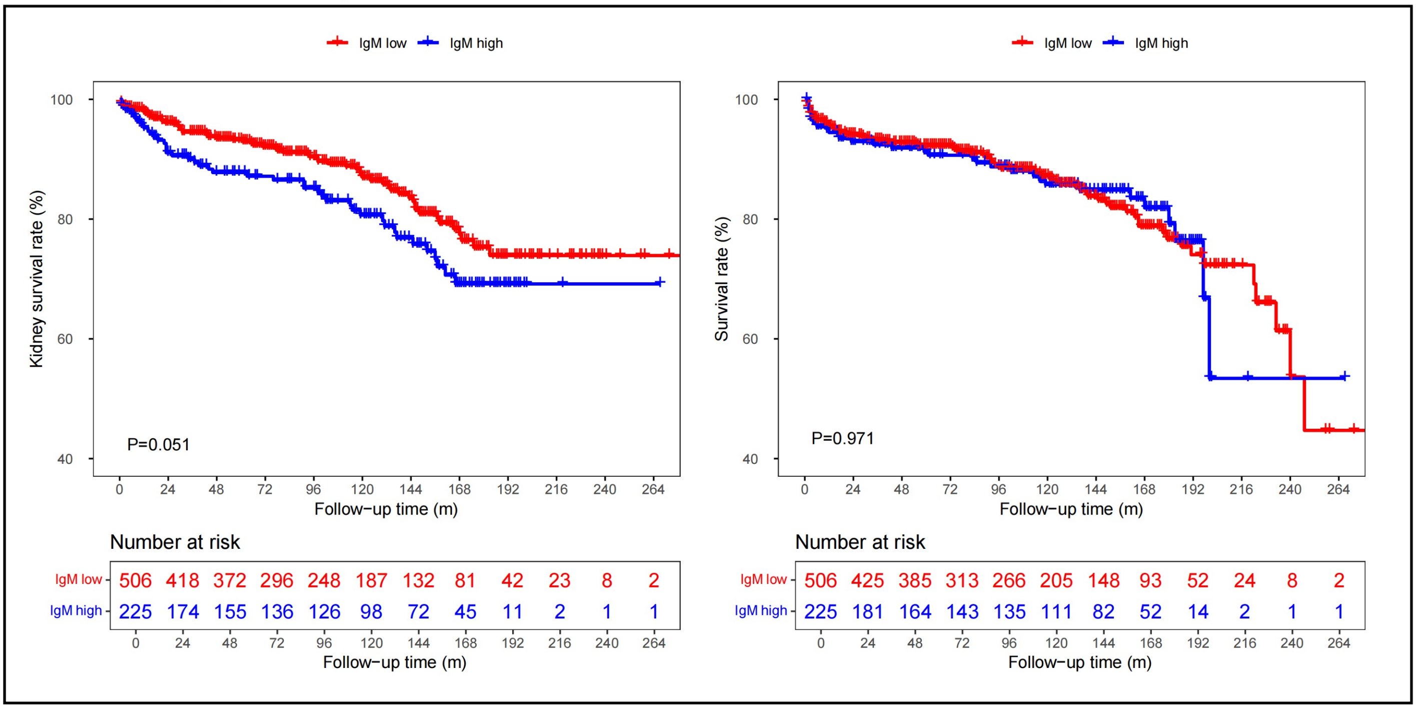

(IgM low=− to +; IgM high = ++ to ++++)

Figure S7. Prognostic impact of glomerular IgM deposition on renal and overall survival in lupus nephritis patients with low C3/C1q deposition.

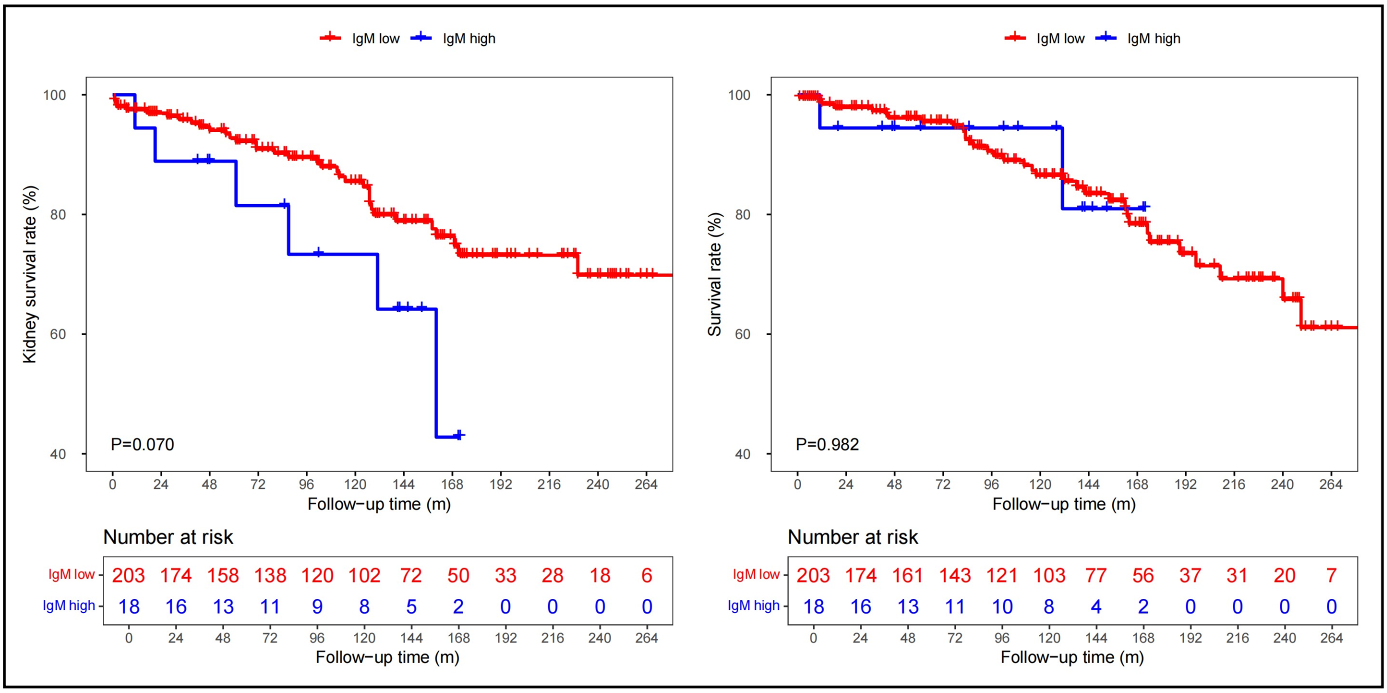

Supplement: online supplemental file 2 [file lupus-12-2-s002.pdf]
